# The promising antioxidant effects of lignans: Nrf2 activation comes into view

**DOI:** 10.1007/s00210-024-03102-x

**Published:** 2024-05-02

**Authors:** Emad H. M. Hassanein, Hanan S. Althagafy, Mohammad A. Baraka, Esraa K. Abd-alhameed, Islam M. Ibrahim, Mostafa S. Abd El-Maksoud, Nesma M. Mohamed, Samir A. Ross

**Affiliations:** 1 Department of Pharmacology and Toxicology, Faculty of Pharmacy, Al-Azhar University, Assiut Branch, Assiut, 71524 Egypt; 2 Department of Biochemistry, Faculty of Science, University of Jeddah, Jeddah, Saudi Arabia; 3 Faculty of Pharmacy, Al-Azhar University, Assiut Branch, Assiut, 71524 Egypt; 4 Department of Pharmacology and Toxicology, Faculty of Pharmacy, Beni-Suef University, Beni-Suef, Egypt; 5 Department of Pharmacognosy, Faculty of Pharmacy, Assiut University, Assiut, 71526 Egypt; 6 Department of Pharmacognosy, Faculty of Pharmacy, Badr University in Assiut, Assiut, 77771 Egypt; 7 National Center for Natural Products Research, Research Institute of Pharmaceutical Sciences, School of Pharmacy, The University of Mississippi, University, MS 38677 USA; 8 Department of BioMolecular Sciences, Division of Pharmacognosy, School of Pharmacy, University of Mississippi, University, MS 38677 USA

**Keywords:** Lignans, Antioxidant, Nrf2/ARE, Inflammation, Phytochemicals

## Abstract

Lignans are biologically active compounds widely distributed, recognized, and identified in seeds, fruits, and vegetables. Lignans have several intriguing bioactivities, including anti-inflammatory, antioxidant, and anticancer activities. Nrf2 controls the expression of many cytoprotective genes. Activation of Nrf2 is a promising therapeutic approach for treating and preventing diseases resulting from oxidative injury and inflammation. Lignans have been demonstrated to stimulate Nrf2 signaling in a variety of in vitro and experimental animal models. The review summarizes the findings of fourteen lignans (Schisandrin A, Schisandrin B, Schisandrian C, Magnolol, Honokiol, Sesamin, Sesamol, Sauchinone, Pinoresinol, Phyllanthin, Nectandrin B, Isoeucommin A, Arctigenin, Lariciresinol) as antioxidative and anti-inflammatory agents, affirming how Nrf2 activation affects their pharmacological effects. Therefore, lignans may offer therapeutic candidates for the treatment and prevention of various diseases and may contribute to the development of effective Nrf2 modulators.

**Supplementary Information:**

The online version contains supplementary material available at 10.1007/s00210-024-03102-x.

## Introduction

Recent studies have focused on identifying polyphenols and their derivatives in various biofluids concerning possible health-promoting characteristics (Rothwell et al. 2016). Polyphenols are secondary metabolites in a wide range of vegetables and fruits (González-Vallinas et al. 2013). Because of their antioxidative characteristics, polyphenols are known to prevent disease, slow its progression, and even support the healing process (Ramos 2008; González-Vallinas et al. 2013).

### Chemistry and appearance of lignans

Lignons are a group of secondary metabolites found in plants first identified by Haworth as polyphenols (Teponno et al. 2016; Haworth 1942). Due to their steroid-analogous chemical structure, lignans are recognized as phytoestrogens and play a role in the prevention and treatment of diseases (Rodríguez-García et al. 2019; Santini et al. 2017). Lignans also have a variety of biochemical properties, such as anti-inflammatory (Pietrofesa et al. 2016; Mohamed et al. 2022), antioxidant (H. Lu and Liu 1992), antitumor (Capilla et al. 2001), and neuroprotective (Q. Wang et al. 2019) properties.

Lignans are present in over 70 plant groups. Numerous lignan ingredients exist in the *Lauraceae*, *Annonaceae*, *Orchidaceae*, *Berberidaceae*, and *Schisandraceae* families (Pan et al. 2009; Kaplan 1942; X. Q. Wu et al. 2019c; J. Zhang et al. 2014). Vegetables and fruits, beans, whole grain grains, and oilseeds are the main sources of dietary lignans (Landete 2012; Thompson et al. 2006). Sesame and flax seeds are the most abundant sources of lignans in food plant components (Coulman et al. 2005).

Lignan is composed of two phenylpropanoid C6-C3 units connected by extra ether, lactone, or carbon bonds at the β and β’ carbons (Lewis and Davin 1999). Lignans are classified as diphenolic compounds and are derived from the biosynthesis route of shikimic acid (Imai et al. 2006). There are two types of lignans: lignan and neolignan. They are distinguished by the existence or absence of a phenylpropanoid 8,8′-bond between their monomers, as well as by the smaller number and more restricted evolutionary distribution of lignans (Gottlieb 1972). Lignans are categorized based on the type of extra side groups they contain, which can be either aliphatic or aromatic. Furthermore, four families of linear lignans can be distinguished based on their ability to incorporate oxygen into the framework, namely, lignans derived from butane, butanolides, monoepoxylignans, tetrahydrofuran derivatives, bisepoxylignans, and derivatives of 3,7-dioxabicyclo (3.3.0)-octane derivatives. A C-7/C-6" linkage allows for further cyclization, which leads to the creation of a sizable class of molecules known as cyclolignans (Freudenberg and Weinges 1961). Secoisolariciresinol (Seco), syringaresinol (Syr), matairesinol (Mat), lariciresinol (Lari), pinoresinol (Pin), sesamin (Ses), medioresinol (Med), 7′-hydroxymatairesinol, and isolariciresinol are among the diphenolic chemical compounds that produce lignans. Furofuran is categorized as Pin, Med, Syr, Lari, and Ses; dibenzylbutyrolactone is assigned to Mat; 9,9′-dihydroxydibenzylbutane is assigned to Seco; and 9,9′-dihydroxyaryltetralin is assigned to Isolariciresinola (Umezawa 2003). They are mostly unbound in nature, although a few are glycosides. The biological functions of lignans come in a wide variety of structurally distinct types (Teponno et al. 2016; Pan et al. 2009).

### Pharmacokinetics of lignans

Lignans are present as both aglycones and glycosides in plants (Smeds et al. 2007). Lignan glycosides are converted by gut flora into enterolignans (enterolactone and enterodiol), which are then absorbed by the gastrointestinal tract and mainly undergo glucuronidation and sulfation in the liver and enterocytes (Hoikkala et al. 2003; Axelson and Setchell 1981). Following oral administration of flaxseed to rats, the majority of the lignan metabolites (other than SDG) were found in the portal vein in their conjugated form (Axelson and Setchell 1981). However, SDG must be deglucosylated in the intestines or enterocytes before conjugation in the enterocytes (SP Borriello et al. 1985a). Additionally, after conjugation, flaxseed and associated lignans are primarily transported through the blood and stored mainly in the gut, liver, prostate, brain, and lung (Murray et al. 2007; Mukker 2013). Furthermore, enterolignans can be found in a variety of body fluids, including plasma, saliva, and prostatic fluid (Pamies et al. 2011).

**Fig. 1:**
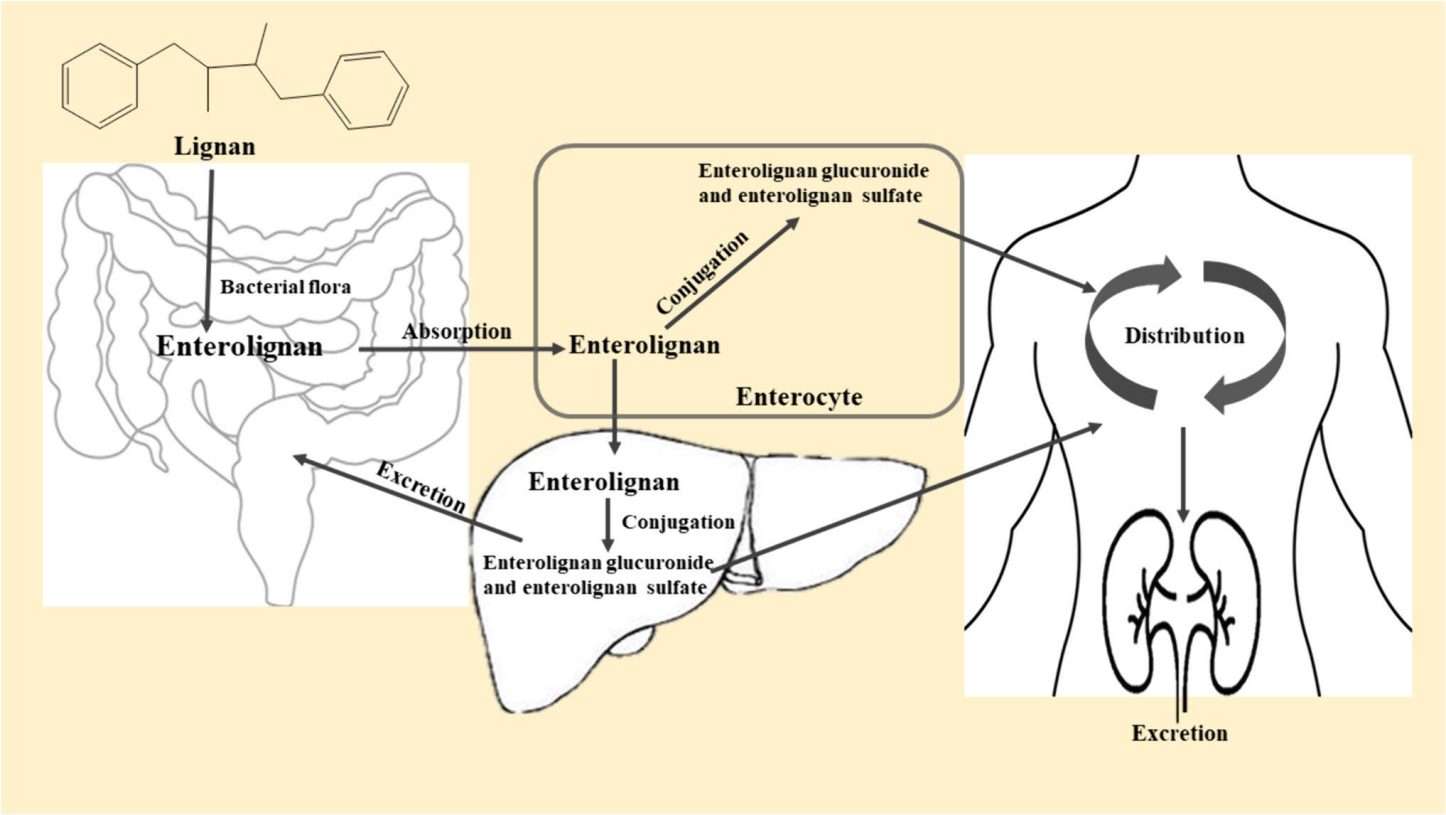
Pharmacokinetics of lignans

The two primary ways lignan metabolites are eliminated in different animals are urine and feces. The consumption of flaxseed increases the number of conjugated enterolignans excreted in the feces (Bach Knudsen et al. 2003). The increased enterolignans in feces may be due to enterohepatic circulation or insufficient absorption. The bile excretes lignan glucuronides, which are subsequently deglucuronidated by bacterial-glucuronidase activity (Jeff Sfakianos et al. 1997a; J. Sfakianos et al. 1997b). However, the majority of the lignans excreted in the urine are eliminated as enterolignan glucuronide, while only a small quantity is eliminated as sulfates and aglycones (Adlercreutz et al. 1995; Axelson and Setchell 1980) (Fig. 1). Several factors influence the hydrolysis required to liberate lignans from sugars, produce enterolignans, and make these compounds bioavailable among individuals due to consumption patterns, altered microbiota, and antibiotic use (Kilkkinen et al. 2001; S. P. Borriello et al. 1985b; Kilkkinen et al. 2002). The pharmacokinetics of specific lignans are provided as a supplementary file (Supplementary 1).

### Pharmacodynamic lignans

Since lignans are phenolic and phytoestrogen substances, numerous studies have investigated their therapeutic potential through various pathways (J. Sfakianos et al. 1997b; Jang et al. 2022). The antioxidant effect has been well-established as an important pathway for the protective and disease-prevention properties of lignans (Kitts et al. 1999). Lignans increase the amounts of catalase (CAT), superoxide dismutase (SOD), and glutathione (GSH), which serve as antioxidants in tissues and protect cells from oxidative damage caused by reactive oxygen species (ROS) (S. P. Ip and Ko 1996b; You and Cho 2021; Nagappan et al. 2018). In addition, the upregulation of antioxidant signals such as heme oxygenase-1 (HO-1), nuclear factor erythroid 2-related factor 2 (Nrf2), and Kelch-like ECH-associated protein 1 (Keap1) by lignans has also been reported (Jiang et al. 2016; Côrtes et al. 2012; V. K. Bajpai et al. 2017b). Furthermore, the anti-inflammatory effect of lignans plays an important role in organ protection. It has been reported that dietary lignans and metabolites regulate inflammation by lowering proinflammatory mediators and suppressing the nuclear factor-κB (NF-κB), MAPK, and JAK/STAT3 signaling pathways (V. K. Bajpai et al. 2018; H. Q. Wang et al. 2022). Lignans also have antiangiogenic, antiproliferative, and anticancer effects in various cancers by regulating matrix metalloproteinases, m-TOR, EGRF, VEGF, and steroid receptor coactivator-3 (Dabrosin et al. 2002; Rauf et al. 2018). Because of their estrogenic potential, lignans have an anti-menopausal effect and decrease the risk of breast cancer (Velentzis et al. 2009).

In addition, a clinical study revealed that consuming lignans over the long term had significant effects on reducing women’s and men’s risk of coronary heart disease over the long term (Hu et al. 2021). This result is in accordance with the effects of lignans on heart disease associated with menopause. Foods high in lignans have been shown to lower lipid oxidation, total cholesterol, and triglyceride levels, which can help to avoid cardiovascular disease and lessen hepatic inflammation caused by a high-fat diet (Nakamura et al. 2020; Xiao et al. 2021). Moreover, lignans have antiosteoporosis (Choi et al. 2014), antifungal (K. M. Li et al. 2019), anti-asthmatic (Iwasaki et al. 1996), and antiviral (Charlton 1998) effects.

## Nrf2 signaling pathway

### Nrf2 signaling pathway and oxidative stress

Oxidative stress initiates and progresses several diseases (Sies et al. 2017; Gorrini et al. 2013; Z. Chen and Zhong 2014; Luc et al. 2019). Generally, ROS refers to oxygen-containing molecules that have reactive characteristics. They include nonradical compounds such as hydrogen peroxide (H_2_O_2_) as well as free radicals such as superoxide (O^•^_2_) and hydroxyl (HO^•^). These molecules are primarily generated from the oxygen used in different metabolic processes that primarily originate in the endoplasmic reticulum (ER), peroxisomes, and mitochondria (Finkel 2012; Handy and Loscalzo 2012). Since varying ROS levels can cause various biological reactions, modulating intracellular ROS levels is essential for maintaining cellular equilibrium (Cairns et al. 2011; Sena and Chandel 2012). Additionally, ROS can serve as signaling molecules that activate NF-κB signaling and proinflammatory mediators (Gloire et al. 2006). However, biological components such as DNA, proteins, and lipids are damaged by excessive ROS generation. This circumstance results in oxidative stress (Gulcin et al. 2010; Altay et al. 2019).

Antioxidants protect biological systems from the damaging effects of ROS and free radicals (Köse et al. 2015). It was discovered in 1994 that the transcription factor Nrf2 regulates the expression of the beta-globin genes. Thus, it plays a critical role in regulating the production of antioxidants within cells (Moi et al. 1994; Sporn and Liby 2012).

The most significant modulator of Nrf2 signaling is the E3 ligase adaptor Keap1 (Hayes and Dinkova-Kostova 2014; Suzuki et al. 2013). Under normal circumstances, the Keap1-Cullin 3 (CUL3) E3 ligase complex and glycogen synthase kinase 3 (GSK-3) inactivate Nrf2 (Hoeflich et al. 2000). When there is oxidative stress, Keap1 is oxidized, making it incapable of binding to Nrf2. This triggers Nrf2 to become stabilized and enter the nucleus (Cuadrado et al. 2019, 2018). In response to Nrf2 translocation and binding to the antioxidant response element (ARE) transcription factor, antioxidant-related genes such as HO-1, NAD(P)H dehydrogenase quinone 1 (NQO1), and *γ*-glutamyl cysteine ligase modulatory (γ-GCS) are expressed (Radjendirane Venugopal and Jaiswal 1996; R. Venugopal and Jaiswal 1998). The increase in antioxidant enzymes, including superoxide dismutase, catalase, and glutathione, resulted in the cells re-establishing the oxidative/antioxidative balance (Taguchi et al. 2011; Meister 1983). Glutathione is an important antioxidant in human cells, produced by γ-GCS, ubiquinone, and tocopherols, which are effective antioxidants because they are produced in reduced forms by NQO1. HO-1 protects cells from ROS and raises ferritin levels to perform an essential antioxidant role (Consoli et al. 2021).

### Nrf2 signaling pathway and inflammation

**Fig. 2:**
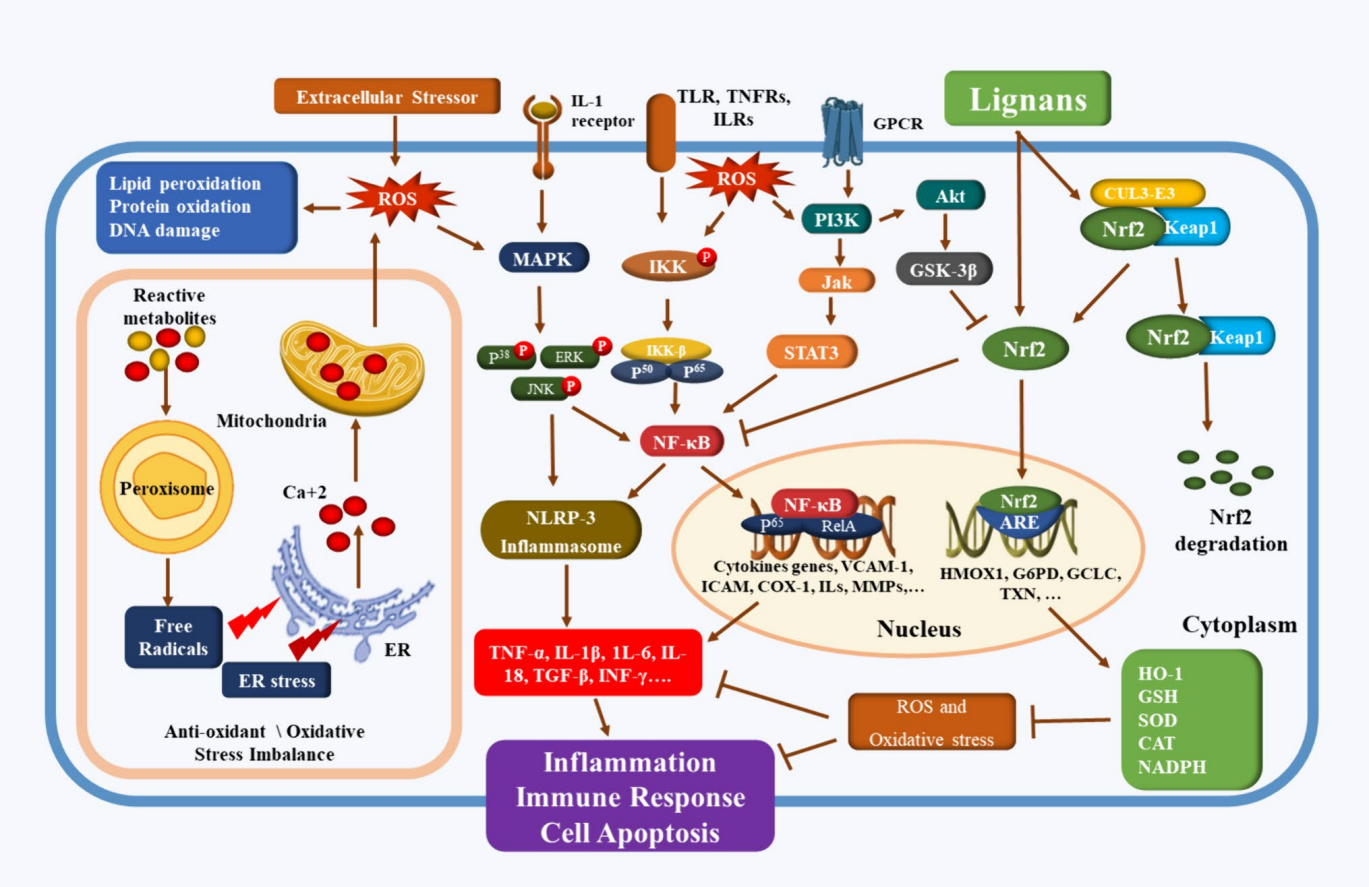
Proposed dual antioxidant and anti-inflammatory effects of lignans by activating Nrf2 signaling pathway. This figure depicts the crosstalk between Nrf2, NF-κB, PI3K, and MAPK signaling and how lignans exert antioxidant and anti-inflammatory effects on modulating these signals and reducing oxidative stress. Increased reactive metabolites, free radicals, and ER stress raise Ca + 2 levels, leading to mitochondrial malfunction and an imbalance in the antioxidant and oxidative stress systems. Increased extracellular stressors and an imbalance in the intracellular antioxidant-oxidative stress system led to an increase in ROS generation, which in turn caused lipid peroxidation, protein oxidation, DNA damage, and the activation of inflammatory signals such as NF-κB, PI3K, and MAPK signaling. In response to the activation of ILRs, TNFRs, and TLRs, as well as elevated ROS levels, normal cells phosphorylate IKK, which activates IKK-B, and the P38, P65 complex, which activates NF-κB. Furthermore, ROS and activation of IL-1β receptor promote P38\MAPK\ERK\JNK signaling, which also activates NF-κB and NLRP-3 inflammasome. ROS and GPCR stimulate PI3K, then activates JAK/STAT3 signaling and NF-κB. Activated NF-κB migrates and translocate into the nucleus, where it binds with P65 and RelA at the binding site on DNA, activating the transcription of inflammatory genes such as cytokines, VCAM-1, ICAM, COX-1, ILs, and MMPs genes. Increased inflammatory gene transcription leads to increased levels of chemokines and cytokines such TNF-α, IL-1β, 1L-6, IL-18, TGF-β, and INF-γ. These cytokines promote inflammation, immunological response, and cell death. On the other hand, the CUL3-E3, Nrf2, Keap1 complex has two ways: it is still connected and results in Nrf2 degradation or it is detached to free active Nrf2, which is how lignans modify this signal. PI3K/AKT/GSK3B signaling reduces Nrf2 activation, whereas active Nrf2 also inhibits NF-κB. The active Nrf2 translocate to the nucleus and binds to ARE on DNA, activating the transcription of HMOX1, G6PD, GCLC, and TXN genes, increasing the levels of antioxidant proteins such as HO-1, GSH, SOD, CAT, and NADPH. These antioxidants prevent cell damage by inhibiting ROS and oxidative stress, which in turn prevents the production of inflammatory cytokines. Abbreviations: ROS; reactive oxygen species, ER; endoplasmic reticulum, ILRs; interleukin receptors; TNFRs, tumor necrosis factor receptors, TLRs Toll like receptors, IL-1; Interleukin-1, MAPK; mitogen-activated protein kinase, JNK; c-Jun N-terminal kinase, ERK; extracellular signal-regulated kinases, IKK; IkappaB kinase, NF-κB; nuclear factor-kappa B, JAK; Janus kinase, STAT3; signal transducer and activator of transcription 3, NLRP-3; NLR family pyrin domain containing-3, TGF-β; tumor growth factor betta, INF-γ; interferon gamma, COX; cyclooxygenase, ICAM; intercellular adhesion molecule, MMP; matrix metalloproteinase, GPCR; G protein coupled receptors, PI3K; phosphoinositide 3-kinase, AKT; protein kinase B, GSK-3β; glycogen synthase kinase 3-betta, Nrf2; nuclear factor erythroid 2-related factor 2, Keep1; Kelch-like ECH-associated protein 1, CUL3-E3; Cullin 3-E3, ARE; antioxidant response element, HO-1; heam oxygenase-1, GSH, reduced glutathione, CAT; catalase, SOD; superoxide dismutase, NADPH; nicotinamide adenine dinucleotide phosphate, G6PD; glucose-6 phosphate dehydrogenase, GCLC; glutamate cysteine ligase catalytic

In addition to upregulating antioxidant gene expression, Nrf2 has a cytoprotective effect by controlling oxidative stress-induced inflammation (J. Ren et al. 2020). NF-κB is a pleiotropic factor that controls several processes, such as cell growth, development, survival, and proliferation, as well as immunity and inflammation (Abdel Moneim 2016). Nrf2 inhibits inflammation by inhibiting NF-κB and releasing proinflammatory cytokines (Q. Ma et al. 2003; W. Li et al. 2008). Nrf2 overexpression suppressed NF-κB activity, which decreased inflammatory cytokine levels in brain, liver, lung, and kidney models (Bhandari et al. 2021; Zeng et al. 2017; Dai et al. 2021; Aly et al. 2017; Hong et al. 2022). It has been shown that the Nrf2 target gene HO-1 blocks the transcription of adhesion molecules by NF-*κ*B, potentially by reducing the amount of free intracellular iron in endothelial cells (Soares et al. 2004). While NF-*κ*B signaling can control Nrf2-mediated antioxidant gene expression via overexpression of the canonical NF-*κ*B component p65, increasing nuclear Keap1 levels results in reduced Nrf2/ARE signaling (M. Yu et al. 2011). Furthermore, it was shown that activating Nrf2 inhibited NF-*κ*B/STAT3 signaling and reduced inflammation (Gong et al. 2020; Hou et al. 2022), and Nrf2 and STAT3 functioned together to control SLC7A11 throughout ferroptosis (Qiang et al. 2020). In addition, Nrf2 was stimulated as an adaptive defense against oxidative stress-induced NLRP3 inflammasome activation initiated by cholesterol crystals (Freigang et al. 2011). As a result of the interaction between Nrf2/keap1 and inflammatory signals such as NF-*κ*B, STAT3, and the NLRP3 inflammasome, Nrf2 activation fundamentally enhances cytoprotection against cellular damaging stimuli, which induce oxidative stress and inflammation (Fig. 2).

However, additional methods for Nrf2 activation have been identified, and these involve kinase pathways such as those of mitogen-activated protein kinases (MAPK) (Rong Yu et al. 1999), phosphatidylinositol-3 kinase (Zheng et al. 2009; Keon Wook Kang et al. 2002), and atypical protein kinase(s) C (Numazawa et al. 2003). Additionally, Nrf2-regulated genes, such as inflammatory regulating and antifibrotic genes, were identified (Hybertson et al. 2011). Therefore, agents that upregulate Nrf2 and its activator will be suggested for further research to treat disorders accompanied by oxidant-antioxidant dysregulation and oxidative stress-induced inflammatory responses.

### Neuroprotective effects of lignans and Nrf2 activation

Several studies reported that Nrf2 activation has a key role in the neuroprotective effects of lignans. Magnolol exhibits a neuroprotective effect in an experimental autoimmune encephalomyelitis model of multiple sclerosis mice. Magnolol treatment significantly decreased oxidative stress, evidenced by decreasing MDA, NO, and MPO while raising the levels of antioxidant enzymes in the brain and spinal cord and reducing inflammatory cell infiltration and proinflammatory cytokines mediated by upregulating the expression of Nrf2 (Bibi et al. 2022). Likewise, magnolol reduced depressive-like behaviors, inhibited proinflammatory cytokines, and increased anti-inflammatory cytokines in chronic unpredictable mild stress-induced depression by upregulating Nrf2 and HO-1 and increasing Nrf2 nuclear translocation (Tao et al. 2021). Also, the protective effects of sesamin on oxidative stress-related neurodegenerative diseases in rat pheochromocytoma PC12 cells were mediated by upregulating HO-1 expression, improving Nrf2 nuclear translocation (Hamada et al. 2011). Additionally, sesamol has an advantageous effect on the liver-brain axis, making it a potential neuroprotective treatment throughout the aging process. Sesamol treatment restored cellular redox equilibrium, protected against mitochondrial dysfunction, and increased antioxidant enzymes in H2O2-treated SH-SY5Y cells by activating Nrf2 and upregulating HO-1 and NQO1(Bo Ren et al. 2018).

### Hepatoprotective effects of lignans and Nrf2 activation

Jiang et al. observed that Schisandrin B (Sch B) could remarkably protect the liver from APAP-induced hepatotoxicity, mainly by activating the Nrf2/ARE signal. Sch B administration increased the expression of Nrf2 downstream GCLC, NQO1, and Nrf2 nuclear accumulation (Jiang et al. 2016). Moreover, Sch B has been shown to treat CCl4-induced liver fibrosis via the Nrf2-ARE and TGF-β/Smad signals. Sch B dramatically reduced collagen accumulation and oxidative stress and inhibited HSC activation by disrupting the TGF-β/Smad signaling pathway (Qingshan Chen et al. 2017). Also, nectandrin B can protect hepatocytes from oxidative stress by activating the Nrf2/ARE pathway, regulated by ERK phosphorylation, and by inactivating GSK-3β in an AMPK-dependent manner (Jae-Sook Song et al. 2016).

### Renoprotective effects of lignans and Nrf2 activation

Huang et al. reported that isoeucommin A derived from *Eucommia ulmoides* Oliv regulated the Nrf2/HO-1 signaling pathway in high-glucose-stimulated human renal mesangial cells and protected renal tubular epithelial cells from H2O2-stimulated oxidative injury. Isoeucommin A might mitigate kidney damage by enhancing the expression of SOD, glutathione, HO-1, and Nrf2 while significantly lowering the levels of TNF-α, IL-1β, IL-6, and MDA by stimulating the Nrf2/HO-1 signal (Qi Huang et al. 2021a).

### Cardioprotective effects of lignans and Nrf2 activation

According to Han et al., Sch B prevents oxidative stress-induced cardiovascular diseases by enhancing Nrf2 expression and significantly decreases angiotensin II-induced oxidative stress, mitochondrial membrane-potential depolarization, and mortality in rat aortic endothelial cells (Han et al. 2018).

## The modulatory effect of lignans on Nrf2

### Schisandra chinensis

*Schisandra chinensis* Turcz (S. chinensis) and *Schisandra sphenanthera* family Schisandraceae are extensively dispersed in China, Japan, and Korea and are recognized for their five-flavor fruits (Hancke et al. 1999; S. Huang et al. 2021b). Schisandra fruit is a common herbal supplement in both Western phytotherapy and traditional Chinese medicine, and it is used to increase disease and stress tolerance, as well as physical performance (Szopa et al. 2017; Nowak et al. 2019; S. Huang et al. 2021b). Schisandra fruit extracts provide a wide variety of therapeutic advantages in both normal and pathological circumstances, including antioxidant, anti-inflammatory, anticancer, antibacterial, antiviral, hepatoprotective, cardioprotective, and hypoglycemic properties (Hancke et al. 1999; Nowak et al. 2019; Szopa et al. 2017; Szopa and Ekiert 2012). *S. chinensis* biologically active compounds have been reported to upregulate antioxidant enzymes while downregulating proinflammatory cytokines and inhibiting apoptosis (Oh et al. 2010; Chiu et al. 2008; Na Chen et al. 2008). Furthermore, ethanolic extract from *S. chinensis* fruit may directly scavenge ROS, lowering the growth inhibition of C2C12 cells induced by H2O2 alleviation and neutralizing ROS generated by phorbol myristate acetate-stimulated human polymorphonuclear leukocytes (Ji Sook Kang et al. 2014; Xiaojie Li et al. 1990).

#### Schisandrin A

Schisandrin A is a dibenzocyclooctadiene derivative derived from *S. chinensis* and *S*. *sphenanthera*. It has been demonstrated to have anti-inflammatory, antioxidant, renoprotective, and neuroprotective properties (Gui et al. 2020; Chuwen Li et al. 2018; Huyke et al. 2007). Schisandrin A, in general, increased the expression of Nrf2, HO-1, and Bcl-2 while decreasing the expression of Keap1, Bax, and caspase-3. This increased the level of GSH while diminishing the amount of MDA (Huijiao Lin et al. 2021).

Shisandrin A activates the Nrf2/HO-1 pathway by inhibiting the MAPK/PI3K/Akt pathway, thus protecting RAW 264.7 cells from LPS-induced inflammation and oxidative stress. Furthermore, schisandrin A effectively increased Nrf2 and HO-1 expression, decreased the LPS-stimulated increase in intracellular ROS, and inhibited macrophage infiltration (Kwon et al. 2018). In this context, Xu et al. observed that Sch A protects against LPS-induced mastitis by activating the Nrf2 signaling pathway and triggering autophagy via the AMPK-ULK1 signaling pathway while reducing the mTOR signaling system. Furthermore, Sch A reduced LPS-induced decreases in proinflammatory and mTOR phosphorylation while activating AMP-activated protein kinase (AMPK) and unc-51-like kinase 1 (ULK1) in murine mammary epithelial cells (Xu et al. 2020). Ni et al. reported that Sch modulates Nrf2 to prevent RANKL-induced ROS in vitro and in vivo to protect against OVX-induced bone loss. Sch promotes Nrf2 expression by inhibiting Nrf2 degradation and suppressing P65 phosphorylation, nuclear translocation, and degradation of IB (Ni et al. 2020).

#### Schisandrin B

Schisandrin B (Sch B) is an active dibenzooctadiene lignan found in the fruit of *S. chinensis* that has antitumor (Zhen Liu et al. 2012), anti-inflammatory (Checker et al. 2012), antioxidant (Siu-Po Ip and Ko 1996a), and neuroprotective (Giridharan et al. 2015) activities. Chen et al. demonstrated that Sch B treatment protects the lungs by increasing Nrf2 activation while decreasing the NF-κB pathway in OVA-induced airway hyperresponsiveness. It also reduced IgE levels, reduced pathological damage, and regulated inflammatory responses (Yaqin Chen et al. 2021). Similarly, Jia et al. reported that Sch B attenuated CS-induced pulmonary inflammation in mice by inhibiting NF-κB activation and increasing Nrf2 and HO-1 expression. Also, Sch B decreased TNF-α, IL-1β, IL-6, MPO, and MDA levels, while it increased antioxidants SOD and GSH (Jia et al. 2017).

Jiang et al. observed that Sch B can remarkably protect the liver from APAP-induced hepatotoxicity, mainly by activating the Nrf2/ARE signal. Sch B administration increased the expression of Nrf2 downstream GCLC, NQO1, and Nrf2 nuclear accumulation (Jiang et al. 2016). Moreover, Sch B has been shown to treat CCl4-induced liver fibrosis via the Nrf2-ARE and TGF-β/Smad signals. Sch B dramatically reduced collagen accumulation and oxidative stress and inhibited HSC activation by disrupting the TGF-β/Smad signaling pathway (Qingshan Chen et al. 2017). The hepatoprotective effects of Sch B include upregulation of CYP450 activity and Nrf2, NQO-1, and GST expression.

Sch B can increase Nrf2-driven thioredoxin expression in alum-induced peritonitis. In RAW264.7 cells, Sch B inhibited LPS/ATP-induced ROS production, JNK1/2 activation, caspase 1 activation, IL-1β release, and LDH production (Leong and Ko 2015). Sch B also activates the Nrf2 signaling pathway to inhibit LPS-induced inflammation in activated human umbilical vein endothelial cells. Sch B inhibited LPS-induced TNF-α, IL-8, ICAM-1, and VCAM-1 expression. Furthermore, it increased the expression of Nrf2 and HO-1 while decreasing the expression of NF-κB (Qiuning Lin et al. 2017). Furthermore, Zhang et al. reported in 2021 that Sch B Sch B reduced colitis-related epithelial cell damage and mitochondrial damage caused by increased ROS by modulating AMPK/Nrf2-dependent signaling and by inhibiting the activity of the NLRP3 inflammasome (Weiwei Zhang et al. 2021a).

Additionally, Sch B promotes neuroprotection and cardioprotection by increasing the expression of the Nrf2 signaling pathway. According to Han et al., Sch B is a Keap1 inhibitor that may be applied to prevent oxidative stress-induced cardiovascular diseases by enhancing Nrf2 expression. Furthermore, Sch B significantly decreased angiotensin II-induced oxidative stress, mitochondrial membrane-potential depolarization, and mortality in rat aortic endothelial cells (Han et al. 2018). Wu et al. (2019a, b, c, d) also demonstrated that enhancing antioxidants via the Nrf2 pathway alleviates anxiety-like behavior (Ying Wu et al. 2019d).

However, Sch B has been studied in vitro as an antioxidant. Chiu et al. (2011) studied how Sch B influences MAPK and Nrf2 activation, glutathione response induction, and apoptotic protection in H9c2 cells. They reported that Sch B activates redox-sensitive ERK/Nrf2 signaling, which results in a glutathione antioxidant response and the prevention of hypoxia/reoxygenation-induced apoptosis (Chiu et al. 2011). It also activates Nrf2/ARE-mediated oxidative response genes such as GCLC, NQO1, and HO-1, accumulating Nrf2 in the nucleus in HK-2 cells exposed to cisplatin-induced oxidative stress (Mei Li et al. 2012). In addition, Dong et al. revealed that the Nrf2/ARE pathway is important in preventing BaP from damaging HTR cells. Sch B upregulated Nrf2, HO1, NQO1, and SOD (Dong et al. 2016). Furthermore, in 2021, Ding et al. studied the ability of Sch B to protect HaCaT cells from oxidative damage caused by tert-butyl hydroperoxide by enhancing the Nrf2 signaling pathway and, as a result, antioxidant enzymes. Sch B significantly reduced DNA damage, protein oxidation, lipid peroxidation, ROS, and cell death while decreasing mitochondrial membrane potential (MMP) and ATP levels (Ding et al. 2018).

#### Schisandrin C

Schisandrin C from *S. chinensis* has considerable therapeutic benefits due to its antioxidant and anti-inflammatory characteristics (Panossian and Wikman 2008; Chun et al. 2014). Han et al. revealed that Sch C treatment improves relaxation and lowers aortic oxidative stress in rats provided subcutaneous Ang II infusions, indicating that Sch C might be used to treat vascular endothelial deficits. They also revealed that Keap1, a negative regulator of Nrf2, is a target of Sch C utilizing an expression plasmid and molecular docking (Han et al. 2019).

### Magnolol

Magnolol (2-(2-hydroxy-5-prop-2-enylphenyl)-4-prop-2-enylphenol), derived from the stem bark of *Magnolia officinalis*, is mostly used in Chinese medicine (Kachur and Suntres 2020). Magnolol is known to have a variety of advantageous pharmacological effects, including anti-inflammatory (Kwak et al. 2012; Weeks 2009), antioxidant (Yung-Hsiang Chen et al. 2009), antibacterial (You-Jin Kang et al. 2012), anti-osteoclastic (Yang et al. 2008), antianxiety (Tsai et al. 2010), antidiabetic (Jong Soon Kang et al. 2008), antiplatelet, and anticarcinogenic (Shih and Chou 2012) properties.

Bibi et al. reported that magnolol exhibits a neuroprotective effect in an experimental autoimmune encephalomyelitis model of multiple sclerosis mice. Magnolol treatment significantly decreased oxidative stress by reducing MDA, NO, and MPO while raising the levels of antioxidant enzymes in the brain and spinal cord. In addition, Magnolol enhances the antioxidant defense mechanism and reduces inflammatory cell infiltration and proinflammatory cytokines by upregulating the expression of Nrf2 and suppressing the expression of iNOS and cleaved caspase-3 (Bibi et al. 2022). Likewise, Tao et al. reported that magnolol reduced depressive-like behaviors, inhibited proinflammatory cytokines, and increased anti-inflammatory cytokines in chronic unpredictable mild stress-induced depression via Nrf2/HO-1/NLRP3 signaling. Magnolol increases the expression of Nrf2 and HO-1, increases Nrf2 nuclear translocation, lowers ROS levels, and decreases the expression of NLRP3, caspase-1, and inflammatory cytokines (Tao et al. 2021).

According to Lu et al. in 2020, magnolol may play a role in the mitigation of periodontitis by suppressing LPS-induced inflammation in macrophages through its activation of HO-1. Magnolol significantly stimulated the Nrf2/HO-1 cascade and the p38 MAPK pathway. Magnolol administration significantly reduced inflammatory responses, as revealed by suppressing proinflammatory cytokines and NF-κB activation, with a substantial increase in Nrf2 nuclear translocation and HO-1 activity (Sheng-Hua Lu et al. 2015). In addition, Liu et al. demonstrated that upregulation of Nrf2 signals improved wound healing and reduced inflammation in diabetic patients with periodontal disease. Magnolol increases the expression of Nrf2 and HO-1, diminishes the levels of IL-6 and IL-8, and attenuates the production of ROS caused by advanced glycation end products (Chia-Ming Liu et al. 2021).

### Sesame oil

Sesame (*Sesamum indicum L*.) is an ancient oilseed crop prevalent in subtropical and tropical areas (Bedigian and Harlan 1986). Sesame oil has antioxidant and health-promoting characteristics due to tocopherols, tocotrienols, and lignans (Pathak et al. 2014; Namiki 2007). Sesame lignans are known for their beneficial effects, which include decreasing blood glucose and cholesterol levels, protecting against cancer and cardiovascular disease, and relieving postmenopausal symptoms (D. Wu et al. 2019a). Sesamin and sesamol are the two main lignans in sesame (Namiki 2007; Majdalawieh and Mansour 2019).

#### Sesamin

Sesamin 4-[(3S,6S)-3-(3,4-dihydroxyphenyl)-1,3,3a,4,6,6a-hexahydrofuro[3,4-c]furan-6-yl]benzene-1,2-diol has been studied extensively for its several potential functions, including hepatoprotective (Ide et al. 2001), antitumor (Hibasami et al. 2000), antihypertensive (Miyawaki et al. 2009), anti-inflammatory (K. Li et al. 2016), and antioxidant (Lei et al. 2012) properties. According to in vivo and in vitro studies by Wang et al. (2021a, b), sesamin significantly mitigates I/R-induced intestinal damage in rats by increasing the expression of Nrf2, HO-1, and NQO1 (Yilin Wang et al. 2021b). In 2019, Kong et al. reported that sesamin on chondrocytes stimulated by IL-1β from human osteoarthritis patients. They demonstrated that sesamin treatment significantly decreased IL-1β-induced PGE2, NO, and MMP13 production. Sesamin also promoted the expression of Nrf2 and HO-1 and inhibited IL-1β from phosphorylating NF-κB p65 and IκB (Kong et al. 2016). Additionally, Bai et al. (2019) investigated the preventive effect of sesamin against dextran-induced UC via the activation of Nrf2-targeted genes. Sesamin protects against UC, and this effect is related to activated AKT/ERK and improved Nrf2 signaling (Bai et al. 2019). Additionally, Hamada et al. observed the effect of sesamin on oxidative stress-related neurodegenerative diseases in rat pheochromocytoma PC12 cells through activating Nrf2/ARE signaling. Sesamin promoted HO-1 expression, improved Nrf2 nuclear translocation, and suppressed p38 MAPK activation and intracellular ROS concentrations (Hamada et al. 2011).

#### Sesamol

Sesamol (1,3-benzodioxol-5-ol) is a lignan with chemopreventive, antioxidant (Kanu et al. 2010), antibacterial, antifungal (Rao et al. 2013), anti-inflammatory (Chu et al. 2010), and neuroprotective (Kanu et al. 2010) properties. According to Ren et al. (2018), sesamol has an advantageous effect on the liver-brain axis, making it a potential neuroprotective treatment throughout the aging process. Sesamol treatment restored cellular redox equilibrium, protected against mitochondrial dysfunction, and increased antioxidant enzymes in H2O2-treated SH-SY5Y cells by activating Nrf2. Furthermore, sesamol notably decreased oxidative stress-induced cognitive impairments, lowered inflammatory cytokines, and upregulated HO-1 and NQO1(Bo Ren et al. 2018).

### Saururus chinensis

*S. chinensis* dried stems have been used for centuries in China, Japan, and Korea and exhibit a variety of biological actions, including anti-inflammatory (Lee et al. 2006), antitumor (Hodges et al. 2004), antioxidant (Rajbhandari et al. 2001), hepatoprotective (Lishu Wang et al. 2009), anticancer (Do et al. 2013), cholesterol-lowering (Yun et al. 2007), and bone protective (Gao et al. 2018) activities. According to Jung et al., *S chinensis* lignans can potentially treat inflammation by modulating the Nrf2/HO-1 signal. *S. chinensis* lignans can inhibit the production of inflammatory cytokines in response to LPS. These lignans were also observed to significantly inhibit LPS-induced production of iNOS, COX-2, IL-6, and TNF-α. Furthermore, these lignans promote the nuclear translocation of Nrf2 and the expression and level of HO-1 (Jung et al. 2019).

The unique lignan Sauchinone isolated from *Saururus chinensis* (Saururaceae) has been shown to exhibit anti-inflammatory (Min et al. 2009), antioxidant (D. Zhou and He 2022), antiapoptotic (Hyun Song et al. 2003), and hepatoprotective (Sung and Kim 2000) characteristics. Kay et al. showed that sauchinone protects the liver from acetaminophen-induced toxicity by activating Nrf2, which is mediated by activated PKCδ, resulting in the inhibitory phosphorylation of GSK3. In addition, sauchinone upregulates Nrf2 expression and enhances the nuclear accumulation of Nrf2 and the production of the reporter gene for the ARE, glutamate-cysteine ligase, and NQO1 protein, all of which assist in restoring the glutathione content of the liver. Sauchinone treatment thereby improved Nrf2 phosphorylation while decreasing its interaction with Keap1. Furthermore, it inhibits GSK3 phosphorylation, activates PKC, and upregulates Nrf2 activity (Kay et al. 2011).

### Pinoresinol

Pinoresinol diglucoside (PDG) is one of the principal lignans extracted from Eucommia ulmoides. According to a previous study, PDG has numerous pharmacological properties, including anti-inflammatory (During et al. 2012), antioxidant (Tebboub et al. 2018), antihypertensive (L. F. Luo et al. 2010), and tumor‐suppressive (Horn-Ross et al. 2003) properties. According to Zhang et al., PDG inhibits inflammatory and oxidative damage caused by I/R in a mouse model of middle cerebral artery obstruction. PDG treatment dramatically increased the activities of SOD, GSH-Px, and GSH and decreased lipid peroxidation while significantly decreasing the levels of the cytokines TNF-α, IL-1β, and IL-6 in mouse brain tissue. PDG may also promote Nrf2 and HO-1 expression and suppress NF-κB activation (Yi Zhang et al. 2021b).

### Phyllanthin

In the plant extract of the *Phyllanthus amarus* family of the *Euphorbiaceae*, phyllanthin is a significant bioactive lignan component that has been extracted and has been shown to have antibacterial action (Adegoke et al. 2010). Phyllanthin has been shown to have anti-inflammatory (Harikrishnan et al. 2018), hepatoprotective (Krithika et al. 2015), antitumor (H. Wang et al. 2021a), antibacterial (Upadhyay et al. 2010), and antioxidant (Chirdchupunseree and Pramyothin 2010) properties.

Yuan et al. investigated how phyllanthin protects the ischemic brain, suggesting its use in treating CIR and associated damage. CIR inhibited the expression of Nrf2, AMPK, and NF-κB, indicating that it stimulates crosstalk between the NF-κB and Nrf2 pathways. However, it increased IL-10 levels in chemokines, lowered inflammatory cytokines, and increased antioxidative enzyme activity (Yuan et al. 2021). Additionally, Wu et al. demonstrated that phyllanthin and hypophyllanthin decreased the Th2 response in mice induced with OVA-induced asthma. A significant reduction in SOD, NO, GSH, and MDA was observed by PA, as well as a significant reduction in OVA-induced increases in IgE levels. As a result of PA treatment, there was a significant reduction in the levels of the Th2 cytokines IL-4 and IL-6, as well as immune-inflammatory markers such as IL-1β, TNF-α, and TGF-β, as well as Nrf2, HO-1, and iNOS, which were elevated (Wei Wu et al. 2019b).

### Nectandrin B

Nutmeg is the dried extract of the *Myristica fragrans* Houtt plant and has long been used as a spice as a remedy for diarrhea (Nguyen et al. 2010; Van Gils and Cox 1994). It has also been shown to have anti-inflammatory, antihyperlipidemic, and antiatherosclerotic effects (Olajide et al. 1999; Ram et al. 1996). Nectandrin B is a nutmeg extract that exhibits antioxidant and anti-inflammatory properties as well as a powerful activating impact on AMPK signaling (Nguyen et al. 2010; Nakano et al. 2017). Also, nectandrin B can protect hepatocytes from oxidative stress by activating the Nrf2/ARE pathway, regulated by ERK phosphorylation, and by inactivating GSK-3β in an AMPK-dependent manner (Jae-Sook Song et al. 2016).

### Isoeucommin A

*Eucommia ulmoides* Oliv contains a wide variety of bioactive compounds, including lignans, which possess powerful antioxidative and anti-inflammatory properties (Fujikawa et al. 2010; D. Luo et al. 2014). Huang et al. reported that isoeucommin A derived from *Eucommia ulmoides* Oliv regulated the Nrf2/HO-1 signaling pathway in high-glucose-stimulated human renal mesangial cells and protected renal tubular epithelial cells from H2O2-stimulated oxidative injury. Isoeucommin A might mitigate kidney damage by enhancing the expression of SOD, glutathione, HO-1, and Nrf2 while significantly lowering the levels of TNF-α, IL-1β, IL-6, and MDA by stimulating the Nrf2/HO-1 signaling process (Qi Huang et al. 2021a). As a result, diabetic nephropathy (DN) models in vitro and in vivo could be reduced due to reduced oxidative stress and inflammation.

### Arctigenin

Arctigenin (AR) is an active compound found in *Arctium lappa* L (A lappa), a well-known medicinal herb and nutritional supplement in traditional medicine (Pharmacopoeia 2010). AR has attracted the interest of investigators due to its anti-inflammatory (Kou et al. 2011), antioxidant (Wen-zhou Zhang et al. 2015), antiviral (Kim et al. 2011), antidiabetic (Y. Zhou et al. 2023), and neuroprotective (Fan et al. 2012) actions. Salama et al. observed whether arctigenin might alleviate cadmium-induced nephrotoxicity by reducing ERS and targeting Nrf2 and NF-κB signaling. Arctigenin promoted the nuclear translocation of Nrf2 and enhanced the activity of the redox-regulating enzymes HO-1 and NQO1. Furthermore, arctigenin inhibited cadmium-induced nuclear translocation of NF-κB and the downstream proinflammatory cytokines TNF-α and IL-1β (Salama et al. 2021).

### Lariciresinol

*Isatis indigotica* (Ban-Lan-Gen) is a prevalent, significant, and popular herbal medicine for the clinic as an antiviral plant (Ye et al. 2011). Lariciresinol is an antioxidant (Vivek K Bajpai et al. 2017a), antiviral (B. Zhou et al. 2017), anticancer (Z. J. Ma et al. 2016), and antidiabetic (Alam et al. 2022) lignan extract from *Isatis indigotica*. A study by Bajpai et al. showed that Lariciresinol inhibits ROS production in RAW 264.7 cells and promotes antioxidant enzyme transcription and translation via Nrf2-mediated HO-1 activation via the p38 signaling pathway. Furthermore, lariciresinol treatment increased the transcriptional and translational activity of Nrf2 and HO-1, which counteracted ROS formation (Vivek K Bajpai et al. 2017a).

## In silico analysis of lignans binding to Keap1

**Fig. 3:**
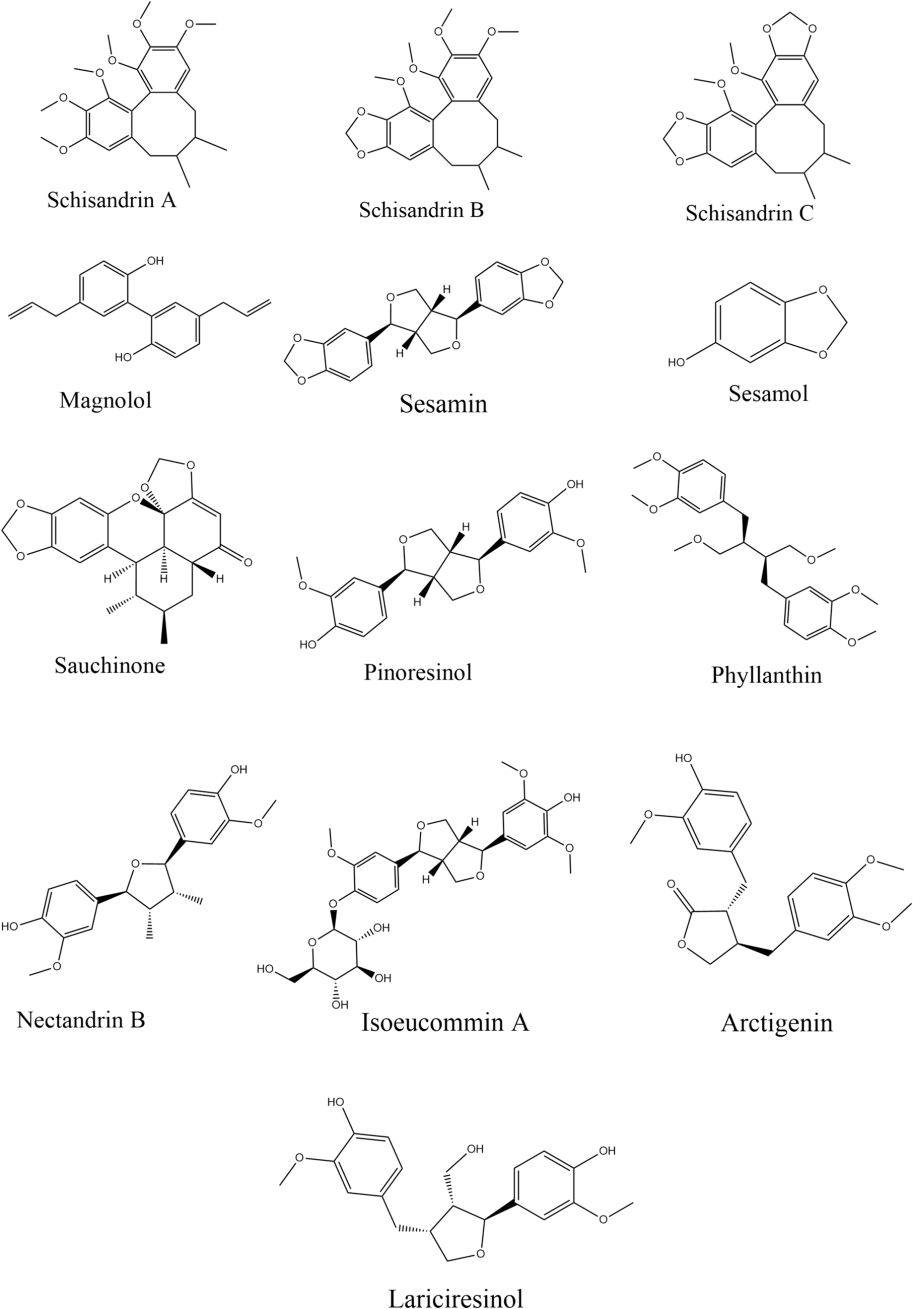
The chemical structures of the selected lignans

A major objective of our research was to provide in silico evidence that lignans bind Keap1, thus potentially serving as Nrf2 activators. In molecular docking (MD), ligands are examined for their binding mechanisms, and their biological properties are confirmed. MD was used to predict ligand binding against targeted proteins at the molecular level using PyRx software (Dallakyan et al. 2015). To determine the binding affinity, the crystal structure of the Keap1 kelch domain was retrieved from PDB ID: 4L7B. As a result of a previous study, the amino acids that interact with the protein were identified (Jnoff et al. 2014). Using AutoDock Tools, all missing atoms were added, Kollman charges were added, and pdbqt files were generated as a result (Morris et al. 2009). The 2D structure of the ligands was sketched using Chamedraw ultra, and Chem3D Pro 12.0 was used to minimize energy and export the pdb files of the ligands (Cousins 2011). The grid box dimensions were set up using PyRx to accommodate all the residues in the protein’s binding pocket. PyRx was also used to produce the required data files. The Discovery Studio visualizer program was used to examine and display the ligand‒protein complexes (Biovia 2017). To validate the MD protocol, we performed redocking of the cocrystallized ligands into the active site of Keap1. According to previous literature, the root mean square deviation (RMSD) value for the redocked structure is < 2.0 Å (Yusuf et al. 2008). After the validation process, we docked our ligands into the active site of the target protein and selected the pose with the lowest binding energy as the optimal pose. The binding energies of the investigated compounds (Fig. 3) are displayed in the following table (Table 1).

**Table 1:** The binding energy of studied lignans to Keap1

Target	Compound	Binding energy (kcal/mol)
Keap1	Standard ((1 s,2r)-2-{[(1 s)-1-[(1,3-Dioxo-1,3-Dihydro-2 h-Isoindol-2-Yl)methyl]-3,4-Dihydroisoquinolin-2(1 h)-Yl]carbonyl}cyclohexanecarboxylic Acid)	− 11.9
	Arctigenin	− 8.5
	Isoeucommin A	− 10.7
	Lariciresinol	− 9.3
	Magnolol	− 7.6
	Nectandrin B	− 9.2
	Phyllanthin	− 7.8
	Pinoresinol	− 9.6
	Sauchinone	− 10.2
	Schisandrin A	− 8.3
	Schisandrin B	− 7.9
	Schisandrian C	− 8.4
	Sesamin	− 10.8
	Sesamol	− 5.9

**Fig. 4:**
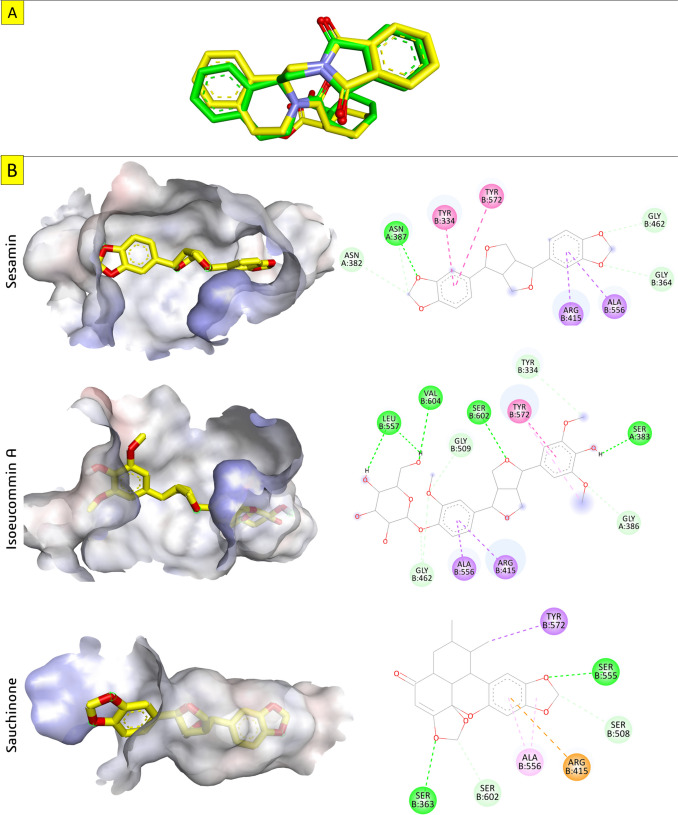
Docking models of sesamin, Isoeucommin A, and Sauchinone. **A** Superimposition of the re-docked (yellow) and co-crystallized (green) in the Keap1 active site. **B** Surface mapping and 3D plot of the promising docked compounds into Keap1. As shown in the figure, sesamin fits properly in the target protein binding pocket by forming a hydrogen bond with Asn387. Additionally, it forms two π-π and two π-sigma interactions with Tyr334, Tyr572, Arg415, and Ala556. Moreover, it forms C–H bonds with Gly364, Asn382, and Gly462. Isoeucommin A binds well in the active site of the protein by forming hydrogen bonds with the key residues of the target protein. These amino acid residues are Ser383, Leu557, Ser602, and Val604. Additionally, it is an aromatic ring that forms one π-π interaction with Tyr572 and two π-sigma interactions with Arg415 and Ala556. Furthermore, it forms C-H bonds with Tyr334, Gly386, and Gly509. All these interactions allow isoeucommin A to bind with Keap1 in a stable manner. Sauchinone interacts with seven active residues in the binding pocket of the target protein. These residues are Ser363, Arg415, Ser508, Ser555, Ala556, Tyr572, and Ser602. Two hydrogen bonds are formed between the oxygen atom of benzodioxole and Ser363 and Ser555

The following table shows the binding features and the favorable interactions of the best-docked pose of the promising compounds with key amino acid residues of Keap1. In addition to their well-fitting properties around the binding pocket, these compounds are suitable candidates for drug development, but we selected the one with the highest binding affinity (Table 2). Sesamin bound to docked Keap1 with a binding energy of − 10.8 kcal/mol. As shown in Fig. 4, sesamin fits properly in the target protein binding pocket by forming a hydrogen bond with Asn387. Additionally, it forms two π-π and two π-sigma interactions with Tyr334, Tyr572, Arg415, and Ala556. Moreover, it forms C-H bonds with Gly364, Asn382, and Gly462 at distances of 3.33, 3.36, and 3.75 Å, respectively. Isoeucommin A binds well in the active site of the protein by forming hydrogen bonds with the key residues of the target protein. These amino acid residues are Ser383, Leu557, Ser602, and Val604. Additionally, it is an aromatic ring that forms one π-π interaction with Tyr572 at a distance of 4.15 Å and two π-sigma interactions with Arg415 and Ala556. Furthermore, it forms C-H bonds with Tyr334, Gly386, and Gly509 with bond lengths of 3.37, 3.53, and 3.43 Å, respectively. All these interactions allow isoeucommin A to bind with Keap1 in a stable manner. Sauchinone exhibits a binding energy value of − 10.2 kcal/mol with Keap1. Sauchinone interacts with seven active residues in the binding pocket of the target protein. These residues are Ser363, Arg415, Ser508, Ser555, Ala556, Tyr572, and Ser602. Two hydrogen bonds are formed between the oxygen atom of benzodioxole and Ser363 and Ser555 with bond lengths of 2.80 and 238 Å, respectively.

**Table 2:** Details of the promising compounds’ interaction with Keap-1 protein

Compound	Binding features
Sesamin	Tyr334, Gly364, Asn382, Asn387, Arg415, Gly462, Ala556, Tyr572
Isoeucommin A	Ser383, Tyr334, Gly386, Arg415, Gly509, Ala556, Leu557, Tyr572 Ser602, Val604
Sauchinone	Ser363, Arg415, Ser508, Ser555, Ala556, Tyr572, Ser602

## Future recommendation

The Nrf2 signaling pathway is essential for preventing oxidative stress and electrophile-induced cell damage. Numerous diseases and disorders have oxidative damage and excessive ROS generation as part of their etiology. Lignans comprise a fairly large class of organic substances with advantageous pharmacological characteristics. Various studies have shown that the therapeutic actions of certain lignans, both in vitro and in vivo, are mediated by Nrf2 activation. Thus, several of the pharmacological and biological actions of lignans may be explained by stimulation of the Nrf2 signaling pathway. Therefore, lignans are an excellent source for finding therapeutic candidates for the treatment/prevention of many disorders and potential leads in developing efficient Nrf2 modulators.

## Supplementary Information

Below is the link to the electronic supplementary material.

Supplementary file1 (DOCX 31 KB)

## Data Availability

No datasets were generated or analysed during the current study.
