# Supplementary material for: The promising antioxidant effects of lignans: Nrf2 activation comes into view
Source: Naunyn Schmiedebergs Arch Pharmacol. 2024 May 2;397(9):6439–58. doi: 10.1007/s00210-024-03102-x (PMC11422461; doi:10.1007/s00210-024-03102-x)
Supplement: Supplementary file 1 — Supplementary file1 (DOCX 31 KB) [file 210_2024_3102_MOESM1_ESM.docx]

Pharmacokinetics of lignans

| Lignan | Pharmacokinetics | References |
| --- | --- | --- |
| Schisandrin A. | In a research trial, rats were orally administered either pure Schisandrin A (Sch A) at a dosage of 14.56 mg/kg or an SA extract equivalent to 14.56 mg/kg of SA monomer. Subsequently, the scientists monitored the fluctuating levels of Sch A in the plasma of the rats over time. The study outcomes indicated specific pharmacokinetic parameters: a half-life (T1/2) of 6.839 hours, a peak concentration (Cmax) of 0.08023 μg/mL, a peak time (Tmax) of 1 hour, an area under the curve (AUC) of 0.2780 μg/mL hr, a mean residence time (MRT) of 3.863 hours, and a clearance (CL) of 13.49 L/hr/kg. | [1] |
|  | Kim et al. investigated the pharmacokinetics of Sch A in rats using both oral administration (150 mg/kg) and intravenous injection (30 mg/kg) of n-hexane soluble extract of Fructus Schisandrae Chinensis (FSC), They reported that after oral administration, the AUC, Tmax, T1/2, and Cmax were 56.5 μg min/mL, 245 min, 1,242 min, and 0.0962 μg/mL, respectively. For intravenous injection, the half-life, AUC, MRT, and CL of SA were 152 min, 22.2 μg min/mL, 89.7 min, and 29.9 mL/min/kg, respectively. | [2] |
| Schisandrin B. | In a study , micronized Sch B particles (10-20 μm) were utilized. Chromatographic separation employed a BDS Hypersil C18 column (50 × 2.1 mm, i.d. 3.5 μm). Detection of schisandrin B and deoxyschizandrin (internal standard) occurred without interference via multiple reaction monitoring mode with positive electrospray ionization. Pharmacokinetic parameters were computed using a noncompartmental method. Gender significantly influenced the area under the concentration-time curve and maximum concentration. Calculated absolute oral bioavailability of schisandrin B was ~55.0% for female rats and 19.3% for male rats. Schisandrin B displayed linear pharmacokinetic properties across tested oral doses (10, 20, and 40 mg/kg). Following oral administration, Sch B exhibited extensive distribution in ovary and adipose tissue. Additionally, minimal urinary, biliary, and fecal excretion of schisandrin B suggested predominant excretion in metabolite forms. | [3] |
| Magnolo | To investigate magnolol's pharmacokinetics and bioavailability, Sprague-Dawley rats received intravenous bolus (20 mg/kg) and single oral dose plus seven doses (50 mg/kg) of magnolol. Blood samples were drawn via cardiopuncture at specific intervals. Organs (liver, kidney, brain, lung, heart) were collected 30 minutes post the 7th oral dose. Serum and tissue specimens underwent HPLC analysis pre- and post-hydrolysis with β-glucuronidase and sulfatase. Results revealed comparable systemic exposure of magnolol glucuronides post intravenous administration; conversely, magnolol sulfates/glucuronides predominated in the bloodstream post oral intake. However, magnolol predominated in liver, kidney, brain, lung, and heart tissues. Liver contained the highest concentrations of magnolol and magnolol glucuronides. In summary, magnolol sulfates/glucuronides prevailed in circulation post oral intake, whereas magnolol predominated in liver, kidney, brain, lung, and heart tissues; notably, the liver exhibited the highest concentrations of magnolol and magnolol glucuronides. | [4] |
| Sesamin | A study involving 48 healthy subjects was conducted to assess the pharmacokinetics and safety of multiple oral doses of sesame lignans, utilizing a single-blind, placebo-controlled, parallel-group design, Participants were randomly allocated into two groups, receiving either 50 mg of sesame lignans or a placebo once daily for a duration of 28 days, Pharmacokinetics of sesame lignans were examined in 10 out of the 24 subjects from the sesame lignans group. No severe adverse effects were noted. Sesamin exhibited peak plasma concentration at 5.0 hours. The primary metabolite, SC-1, reached its peak plasma concentration at 5.0 hours as well and declined swiftly, with a half-life of 2.4 hours. By the seventh day, plasma concentrations of sesamin stabilized. The safety and tolerability of sesame lignans in healthy individuals were affirmed. Pharmacokinetic analysis revealed no accumulation after administering multiple 50 mg doses of sesame lignans. | [5] |
| Sesamol | This paper explores the bioavailability of sesamol in Sprague-Dawley (SD) rats. Following administration of 50 mg/kg sesamol via gastric gavage (p.o.) or intravenous injection, biological fluid samples were collected for analysis. Pharmacokinetic parameters were determined using a noncompartmental model. Additionally, tissue distribution of sesamol (at a dose of 100 mg/kg administered orally) in SD rats was investigated. Concentration changes of sesamol were monitored in various tissues and plasma over a 24-hour period post oral administration. The study revealed an oral bioavailability of sesamol at 35.5 +/- 8.5%. Sesamol demonstrated the ability to cross the blood-brain barrier and undergo hepatobiliary excretion. Conjugated metabolites of sesamol were widely distributed in SD rat tissues, with the liver and kidneys exhibiting the highest concentrations and the brain the lowest. It is suggested that sesamol initially accumulates in the liver before being transported to other tissues such as the lungs, kidneys, and brain. The primary metabolites of sesamol distributed in the lungs and kidneys were identified as glucuronide and sulfate. | [6] |
| Sauchinone | There is limited information available on the subject, with only this limited data currently accessible, In sauchinone pharmacokinetics, there is significant distribution of sauchinone to the liver, where it undergoes extensive metabolism mediated by UGTs, resulting in the formation of numerous sauchinone metabolites. | [7] |
| Arctigenin | Sixteen piglets weighing an average of 30.0 ± 5.0 kg were given either 2.0 mg/kg arctigenin via intravenous injection or 1.0 g/kg Fructus arctii powder orally. Plasma arctigenin levels were assessed using HPLC. Arctigenin followed a two-compartment model with no absorption. Intravenous administration resulted in the following pharmacokinetic parameters: distribution half-life (t 1/2α) of 0.166 ± 0.022 h, elimination half-life (t 1/2β) of 3.161 ± 0.296 h, apparent volume of distribution (V d) of 0.231 ± 0.033 L/kg, clearance rate (CLb) of 0.057 ± 0.003 L/(h.kg), and area under the curve (AUC) of 1.189 ± 0.057 g.h/mL. Oral administration showed the following parameters: absorption half-life (t 1/2ka) of 0.274 ± 0.102 h, t 1/2α of 1.435 ± 0.725 h, t 1/2β of 63.467 ± 29.115 h, V d of 1.680 ± 0.402 L/kg, CLb of 0.076 ± 0.028 L/(h kg), peak time (t max) of 0.853 ± 0.211 h, peak concentration (C max) of 0.430 ± 0.035 g/mL, and AUC of 14.672 ± 4.813 g/mL. Intravenous administration of arctigenin displayed limited tissue distribution, whereas oral administration of Fructus arctii powder exhibited rapid absorption, widespread distribution, and prolonged elimination, suggesting sustained pharmacological effects. | [8] |

1. Mao, S., et al., *Rapid determination and pharmacokinetics study of lignans in rat plasma after oral administration of Schisandra chinensis extract and pure deoxyschisandrin.* 2011. **25**(7): p. 808-815.

2. Kim, Y.J., et al., *Simultaneous determination of nine lignans from Schisandra chinensis extract using ultra‐performance liquid chromatography with tandem mass spectrometry in rat plasma, urine, and gastrointestinal tract samples: Application to the pharmacokinetic study of Schisandra chinensis.* 2014. **37**(20): p. 2851-2863.

3. Wang, Z., et al., *Investigation of pharmacokinetics, tissue distribution and excretion of schisandrin B in rats by HPLC-MS/MS.* Biomed Chromatogr, 2018. **32**(2).

4. Lin, S.P., et al., *Pharmacokinetics, bioavailability, and tissue distribution of magnolol following single and repeated dosing of magnolol to rats.* Planta Med, 2011. **77**(16): p. 1800-5.

5. Tomimori, N., et al., *Pharmacokinetics and safety of the sesame lignans, sesamin and episesamin, in healthy subjects.* Biopharm Drug Dispos, 2013. **34**(8): p. 462-73.

6. Jan, K.C., C.T. Ho, and L.S. Hwang, *Bioavailability and tissue distribution of sesamol in rat.* J Agric Food Chem, 2008. **56**(16): p. 7032-7.

7. You, B.H., E.C. Gong, and Y.H. Choi, *Inhibitory Effect of Sauchinone on UDP-Glucuronosyltransferase (UGT) 2B7 Activity.* Molecules, 2018. **23**(2).

8. He, B., et al., *Pharmacokinetics of Arctigenin and Fructus Arctii Powder in Piglets.* Front Vet Sci, 2019. **6**: p. 235.
